# Novel Therapeutic Approaches and Alternatives to Antibiotic Therapy for Drug-Resistant Intra-Abdominal Infections

**DOI:** 10.3390/antibiotics15080727

**Published:** 2026-07-27

**Authors:** Elena-Adelina Toma, Octavian Enciu, Irina-Mihaela Matache, Andrei Ludovic Porosnicu, Valentin Calu, Adrian Miron, Maliya Delawan, Mohamad Bydon, Mircea Ioan Popa

**Affiliations:** 1 Surgery Department, Carol Davila University of Medicine and Pharmacy, 050474 Bucharest, Romania; elena-adelina.toma@umfcd.ro (E.-A.T.); andrei.porosnicu@umfcd.ro (A.L.P.); valentin.calu@umfcd.ro (V.C.); adrian.miron@umfcd.ro (A.M.); mircea.ioan.popa@umfcd.ro (M.I.P.); 2 Elias Emergency University Hospital, 011461 Bucharest, Romania; 3 Department of Neurological Surgery, University of Chicago, Chicago, IL 60637, USA; irinamihaela.matache@bsd.uchicago.edu (I.-M.M.); maliya.delawan@bsd.uchicago.edu (M.D.); mohamad.bydon@bsd.uchicago.edu (M.B.); 4 The “Cantacuzino” National Medico-Military Institute for Research and Development, 050096 Bucharest, Romania

**Keywords:** antimicrobial resistance, novel antibiotics, nanoparticles, antimicrobial peptides, bacteriophages, intra-abdominal infections

## Abstract

Antimicrobial resistance (AMR) among pathogens involved in intra-abdominal infections (IAIs) represents a critical and escalating clinical challenge. The interconnected nature of antimicrobial resistance, spanning human medicine, veterinary practice, agricultural use and environmental reservoirs, has required coordinated international responses based on the ‘One Health’ principle. This study presents an update on efforts underway worldwide to develop new antibiotics, novel combined antimicrobial agents, and alternatives to classic therapies for IAIs. New antibiotics or compounds with antibacterial activity are currently in various stages of clinical trials, including several fluoroquinolones, beta-lactamase inhibitors, and polymyxin analogues. To reduce the risk of bacterial resistance, various additions to antimicrobial treatments are being explored, such as nanoparticles (NPs), antimicrobial peptides (AMPs), bacteriophages, the CRISPR/Cas system, and probiotics. Each modality offers distinct mechanisms that circumvent established resistance pathways, including multi-target membrane disruption, sequence-specific gene editing, and microbiome restoration. Current preclinical and clinical evidence is synthesized, and key translational barriers, including delivery challenges, safety concerns, regulatory complexity, and the need for IAI-specific pharmacokinetic data are critically examined. In conclusion, the convergence of novel antibiotic agents and non-traditional antimicrobial strategies reviewed herein provides the foundation for a new paradigm in the management of drug-resistant IAIs. The transition from a monotherapy-centric approach to an integrated, multi-modal treatment framework, guided by rapid diagnostics and informed by antimicrobial stewardship, will be essential to preserve therapeutic efficacy against AMR threats of the coming decades.

## 1. Introduction

The recognition of antimicrobial resistance as a global health emergency was underlined in May 2015 with the approval by the World Health Assembly of the Global Action Plan on Antimicrobial Resistance [1]. This landmark document arose from the growing awareness that the systematic misuse and overuse of antimicrobial agents in human medicine and food production endangered all nations, while few other treatment options were under development. The Assembly recognized that without harmonized and immediate action on a global scale, the world was facing a post-antibiotic era in which common infections could once again become fatal [2].

The Global Action Plan set out five interconnected strategic objectives that continue to guide international efforts. The first objective is to boost awareness and understanding of antimicrobial resistance through targeted communication, education, and training for health professionals, policymakers, and the public. Changing prescriber and patient behavior is vital for lasting progress against antibiotic resistance. The second objective is to strengthen the knowledge base via enhanced surveillance and coordinated research. Effective data collection and analysis are crucial for tracking trends, understanding resilience mechanisms, and detecting emerging threats, yet these capabilities are often limited in many countries [3]. Reducing the incidence of infections by improving sanitation, hygiene, and increasing prevention and control measures is the third objective. The fourth objective aims to optimise the use of antimicrobials in both human and animal health through management programmes, evidence-based guidance, and appropriate regulatory frameworks. This objective recognises that even when antimicrobials remain effective, their misuse accelerates the development of resistance. Finally, the fifth objective calls for the development of economic arguments in favour of sustainable investments in new medicines, diagnostic tools, vaccines and other interventions by local, national and international bodies in the medical field.

Intra-abdominal infections warrant distinct and focused analysis as a clinical priority for several convergent reasons that differentiate them from other systemic infections. First, IAIs represent a unique anatomical and microbiological challenge characterized by polymicrobial communities comprising both aerobic Gram-negative organisms and anaerobes, requiring broad-spectrum coverage and complicating selective therapeutic approaches. Secondly, the peritoneal cavity presents distinct pharmacokinetic barriers that differ substantially from systemic or pulmonary infections and limit the applicability of therapies proven effective in other contexts. Moreover, IAIs carry disproportionately high morbidity and mortality even in developed healthcare systems. Despite source control and optimal antimicrobial therapy, mortality rates for severe intra-abdominal sepsis can reach 70%, and this burden falls most heavily on critically ill patients with multidrug-resistant pathogens for whom treatment options are increasingly exhausted [4,5]. Healthcare-associated IAIs, including those complicating major surgery, traumatic injury, or critical illness, demonstrate resistance rates to conventional antibiotics exceeding 30–50% in many institutional settings, creating a clinical crisis where many commonly used agents are no longer reliable [6]. The presence of multi-drug (MDR), extended-drug (XDR) and pan-drug resistant (PDR) pathogens is associated with higher rates of treatment failure, prolonged hospitalisation, and higher overall mortality, which requires a reassessment of current empirical antimicrobial protocols. Finally, the availability of novel antimicrobial strategies represents an emerging opportunity unique to the current era. Several new antibiotics have recently received regulatory approval specifically for complicated IAIs, while bacteriophage therapy, antimicrobial peptides, nanoparticle-based delivery systems, microbiome-based interventions, and CRISPR-Cas technologies have progressed from theoretical concepts to preclinical validation and early clinical exploration.

This narrative review aims to synthesize evidence on recently approved and emerging antimicrobial agents for cIAIs and characterizes mechanisms of action, preclinical and clinical evidence, spectrum of activity, advantages, limitations, and realistic timelines for clinical implementation of each approach.

## 2. Results

### 2.1. Etiologies and Procedure-Related Sources of IAIs

Community-acquired IAIs arising from appendicitis, diverticulitis, and hollow viscus perforation remain the most common presentations [7]. A substantial proportion of cIAIs are postoperative in origin [8,9]. Hepatobiliary and pancreatic resections carry among the highest rates of organ/space surgical site infection (SSI) of any surgical discipline, with intra-abdominal infectious complications occurring in up to 35% of pancreaticoduodenectomies and 10% of hepatectomies, frequently involving MDR organisms [10,11]. Bariatric surgery, now performed in hundreds of thousands of patients annually, is complicated by peritonitis secondary to anastomotic fistula or staple line leak in 1–7% of cases, with a microbial profile that includes *Candida* species, *Klebsiella pneumoniae*, and *Pseudomonas* spp. [12,13,14]. An increasingly recognized but underappreciated source of cIAIs is spine surgery, particularly procedures employing anterior and lateral approaches to the lumbar spine. Anterior lumbar interbody fusion (ALIF) carries a bowel injury rate of approximately 0.25% and an overall exposure-related complication rate of 4.8% [15]. Lateral interbody fusion (LIF) procedures, including XLIF and OLIF, may also involve injury to retroperitoneal and intra-abdominal structures [16]. Beyond direct iatrogenic injury, spinal infections themselves, including vertebral osteomyelitis, spondylodiscitis, and postoperative implant infections, frequently extend into the retroperitoneal compartment as psoas or iliopsoas abscesses. Among post-spinal surgery infections, *S. aureus* accounts for 40–45% of isolates, with Methicillin-resistant *Staphylococcus aureus* (MRSA) rates of 8–34% depending on the series, while Enterobacterales comprise 30–35% of pathogens, with 23% demonstrating multidrug resistance [17,18].

### 2.2. Novel Agents and Beta-Lactam/Beta-Lactamase Inhibitor Combinations

The past decade has witnessed the approval of several novel antibiotics and beta-lactam/beta-lactamase inhibitor (BL/BLI) combinations that have expanded the therapeutic armamentarium against MDR Gram-negative pathogens implicated in IAIs. Ceftolozane/tazobactam and ceftazidime/avibactam were the first of these next-generation BL/BLI combinations to receive regulatory approval, demonstrating non-inferiority to meropenem in Phase 3 trials for cIAIs and providing enhanced coverage against ESBL-producing Enterobacterales and some carbapenem-resistant strains [19]. Meropenem/vaborbactam, incorporating the novel cyclic boronate inhibitor vaborbactam, extended coverage to *Klebsiella pneumoniae* carbapenemase (KPC)-producing organisms, while imipenem/cilastatin/relebactam (Recarbrio), approved for both cIAIs and complicated urinary tract infections (cUTIs), demonstrated efficacy against KPC-producing Enterobacterales and MDR *Pseudomonas aeruginosa* in the RESTORE-IMI Phase 3 clinical program [20,21]. The 2024 Surgical Infection Society guidelines have recommended imipenem/cilastatin/relebactam as empiric therapy for high-risk IAI patients, reserving it for situations requiring broader-spectrum coverage [22]. Cefiderocol, a siderophore cephalosporin that exploits active iron transport systems to penetrate the bacterial outer membrane, provides coverage across all four Ambler classes of beta-lactamases and has demonstrated activity against carbapenem-resistant Enterobacterales (CRE), carbapenem-resistant *A. baumannii* (CRAB), and MDR *P. aeruginosa*, although emergence of resistance during therapy remains a concern [23]. Ceftobiprole medocaril, a fifth-generation cephalosporin approved in 2024 with dual anti-MRSA and anti-Pseudomonal coverage, and gepotidacin, a first-in-class triazaacenaphthylene topoisomerase inhibitor active against ESBL-producing organisms, represent additional novel scaffolds, although their current approved indications are limited to skin and soft tissue infections, ventilator and hospital-acquired pneumonia, and urinary tract infections, respectively [24,25].

More recently, several BL/BLI combinations targeting the critical unmet need of metallo-beta-lactamase (MBL)-producing Gram-negative bacteria have entered advanced clinical development. Sulbactam/durlobactam (Xacduro), approved in 2023 for hospital-acquired and ventilator-associated bacterial pneumonia caused by Acinetobacter baumannii-calcoaceticus complex, represents a critical advance against CRAB, an organism increasingly encountered in nosocomial IAIs and for which therapeutic options were previously limited to polymyxin-based regimens [26]. Particular interest has been shown towards CRAB strains, with 13 antibiotics under clinical development, some of which have an innovative mechanism of action. Among agents specifically targeting CRAB, the clinical pipeline includes several conventional antibiotic candidates distributed across early and late development. Phase I agents include R-327, xeruborbactam/QPX-7728, upleganan, MRX-8, QPX-9003, zifanocycline/KBP-7072, apramycin/EBL-1003, zosurabalpin/RG-6006, and ANT-3310. BV-100 and OMN-6 have progressed to Phase II, whereas zidebactam/WCK-5222 and funobactam/XNW-4107 are being evaluated in Phase III trials. In parallel, non-traditional CRAB-directed approaches are also advancing through early clinical development (phase 1 or 2), including monoclonal antibodies (TRL-1068, CMTX-101), phage therapy (Phagebank), immunomodulation with recombinant human plasma gelsolin (Rhu-pGSN), microbiome modulation (SER-155), and a genetically modified cationic antimicrobial peptide (PLG-0206) [27].

Aztreonam/avibactam, which exploits the inherent stability of the monobactam aztreonam to MBL-mediated hydrolysis while avibactam provides coverage against co-produced serine beta-lactamases, received FDA approval in 2025 for the treatment of serious Gram-negative infections [28]. Taniborbactam is a novel inhibitor of beta-lactamase (classes A, C and D) and metallo-beta-lactamase in phase 3 studies with promising results [29]. The combination of cefepime and taniborbactam is active in vitro against the majority of carbapenem-resistant Enterobacterales (CRE) and carbapenem-resistant *Pseudomonas aeruginosa* (CRPA) isolates, including carbapenemase-producing CRE and CRPA. Cefepime/taniborbactam represents the first BL/BLI combination to incorporate a direct MBL inhibitor and demonstrated non-inferiority to meropenem in the Phase 3 CERTAIN-1 trial for cUTIs [30,31]. Zidebactam and nacubactam are novel beta-lactam enhancers that function as diazabicyclooctane inhibitors, which not only inhibit beta-lactamases, but also exhibit intrinsic antibacterial activity by inhibiting penicillin-binding protein 2 (PBP2), while the cefepime-zidebactam and meropenem-nacubactam combinations are currently being evaluated as therapeutic alternatives for pathogens resistant to newer agents, such as cefiderocol [32,33]. Cefepime/zidebactam was approved by the FDA in June 2026 for cUTIs, after promising results in a Phase 3 trial (NCT04979806), with pharmacokinetic data supporting potential efficacy in nosocomial pneumonia and abdominal infections [34,35]. Xeruborbactam is a distinct class of boronic acid-derived inhibitors, currently under clinical evaluation, designed to complement beta-lactam antibiotics by targeting a broad spectrum of serine-beta-lactamases [34,36]. Similarly, ANT3310 is being developed as a novel boron-based inhibitor to address the limitations of current therapies, although these agents collectively remain inactive against metallo-beta-lactamase-producing strains [37,38]. Pipeline agents of note include cefepime/nacubactam and aztreonam/nacubactam (both in Phase 3 trials), imipenem/cilastatin/funobactam (Phase 3), and the novel combination of cefiderocol/xeruborbactam, which aims to address class A through D beta-lactamases (currently in Phase 1 drug interaction studies) [34,39]. However, the cefiderocol/xeruborbactam combination proved inferior to cefiderocol/taniborbactam in one study assessing the in vitro activity against cefiderocol-resistant New-Delhi Metallo-beta-Lactamase (NDM)-producing *P. aeruginosa* [40].

This persistent gap in activity against metallo-beta-lactamases underscores the urgent need to continue research into broad-spectrum inhibitors capable of addressing this unaddressed global threat [29,41]. Recent advances in medical biochemistry have focused on improving inhibitor affinity for enzymes and the stability of their complexes, based on the concept of competitive inhibitor design, to provide new directions for the further development of beta-lactamase inhibitors [42].

Beyond the BL/BLI class, eravacycline, a synthetic fluorocycline approved specifically for cIAIs, has demonstrated non-inferiority to meropenem and provides activity against ESBL-producing organisms, though clinical data against CRE remain limited [43]. Several fluoroquinolones, including avarofloxacin, delafloxacin, finafloxacin, zabofloxacin, and non-fluorinated nemonoxacin, are in various stages of clinical trials. These compounds demonstrated superior antibacterial activity, including against ciprofloxacin-resistant pathogens, and were found to be well tolerated by both oral and parenteral administration [44]. Plazomycin is a novel semi-synthetic aminoglycoside derived from sisomycin, active against MDR Enterobacterales, including those producing ESBLs, carbapenemases and aminoglycoside-modifying enzymes, with the exception of AAC(2)-Ia and AAC(2″)-Iva [45]. The FDA has authorized the use of plazomycin to treat complicated urinary tract infections, including pyelonephritis, caused by multidrug-resistant bacterial strains [46]. Plazomycin has demonstrated in vitro activity against MDR bacteria and is among the promising treatment options for complicated intra-abdominal infections. Sulopenem, a synthetic thiopenem first developed by Pfizer (New York, NY, USA) in the 1990s, and its derivative, sulopenem etzadroxil, are being developed as treatments for Gram-negative infections by Iterum Therapeutics (Dublin, Ireland) [47]. A randomized phase 3 study of sulopenem compared to ertapenem in patients with cIAI concluded the non-inferiority of this novel molecule, with the advantage that the de-escalation of treatment with the transition from its intravenous to oral administration was achieved without incident, which represents a success for the management of critically ill patients to achieve the transfer of care from the ICU to the surgical wards, or even discharge them at home, with outpatient medical care until the end of the indicated antibiotic treatment [48].

Several new polymyxin analogues have been developed, showing promising pharmacological activity. Of these, upleganan (also referred to as SPR206 or EVER206) demonstrates high efficacy against Enterobacterales, *Pseudomonas aeruginosa* and *Acinetobacter baumannii*, associated with a significantly reduced toxicity profile compared to polymyxin E (colistin), in the phase 1 studies conducted to date [49,50]. Another analog, MRX-8, developed by MicuRx (Hayward, CA, USA and Shanghai, People’s Republic of China), demonstrated in phase 1 studies that it is an effective alternative for infections with MDR Gram-negative bacteria, and was superior to polymyxin B against infections with carbapenem-resistant *K. pneumoniae, P. aeruginosa* and *E. coli* [51].

### 2.3. Alternatives to Classic Antibiotic Therapies

Over the past decade, in response to the AMR crisis, antibiotic-free therapeutic strategies have been proposed, including nanoparticles and polymer-based antimicrobial delivery systems, antimicrobial peptides, bacteriophages, CRISPR/Cas systems, microbiome-based alternatives, antibodies, or the toxin–antitoxin system [52]. These innovative ways work as suitable substitutes for conventional antibiotics, providing comparable capabilities to manage bacterial infections without exacerbating the problem of antibiotic resistance.

#### 2.3.1. Nanoparticle- and Polymer-Based Antimicrobial Delivery Systems

Metallic and polymeric nanoparticles are a class of antimicrobial agents with strong activity against drug-resistant bacteria and fungi. Silver nanoparticles (AgNPs) demonstrate broad-spectrum bactericidal activity via several concurrent mechanisms. These include compromising the integrity of bacterial cell membranes, inducing the formation of reactive oxygen species (ROS), and impeding both DNA replication and protein synthesis [53]. This multi-target mechanism of action makes the development of resistance much more difficult compared to conventional single-target antibiotics. Zinc oxide (ZnO) and copper oxide (CuO) nanoparticles demonstrated significant in vitro activity against ESBL-producing *E. coli* and MDR *K. pneumoniae*, both of which are predominant pathogens in complicated IAI [54]. Polymeric nanoparticles, including chitosan and polylactic-glycolic acid (PLGA)-based nanocarriers, offer additional advantages as targeted drug delivery vehicles, allowing for the controlled release of encapsulated antibiotics at the site of intra-abdominal infection while minimizing systemic toxicity [55].

The translational relevance of localized antimicrobial delivery is further illustrated in spine surgery. Intrawound vancomycin powder has been widely adopted as a prophylactic strategy [56]. Antibiotic-eluting implant coatings represent a more sophisticated approach: a novel biodegradable polyethylene glycol-polypropylene sulfide (PEG-PPS) polymer coating with both passive vancomycin elution and bacteria-triggered active release significantly reduced spinal implant infection burden in a murine model, while 3D-printed titanium cages with polyvinyl alcohol (PVA)-vancomycin coatings demonstrated sustained bactericidal activity exceeding seven days with effective inhibition of *S. aureus* biofilm in vivo [57,58]. Nanoparticle-based delivery systems may further improve on these approaches by providing sustained release above the minimum inhibitory concentration over extended postoperative windows, enhanced biofilm penetration through their nanoscale dimensions, and more predictable local pharmacokinetics [59,60]. Silver nanoparticle-coated pedicle screws have already demonstrated significant reduction in MRSA biofilm formation in a rabbit spinal implant model [61]. PLGA nanoparticle-loaded bone grafts using hydrophobic ion-paired antibiotics have achieved sustained local antibiotic release, with vancomycin maintained above inhibitory concentrations through day 7 and gentamicin released linearly through day 22, addressing the rapid initial release seen with unformulated antibiotic-loaded bone grafts [59].

Nanoparticles exhibit broad-spectrum activity and the ability to penetrate biofilms, although their clinical application is tempered by concerns about potential toxicity and stability, accumulation in organs, and long-term safety.

#### 2.3.2. Antimicrobial Peptides

Antimicrobial peptides (AMPs) represent another promising class of agents, as naturally occurring or synthetically engineered short cationic molecules functioning as polyamides that exert strong antimicrobial activity through mechanisms distinct from traditional antibiotics [62]. AMPs exert rapid bactericidal activity primarily by interacting electrostatically with the negatively charged bacterial cell membrane, leading to pore formation, membrane depolarization, and cell lysis [63]. Additional intracellular mechanisms include inhibition of cell wall synthesis, nucleic acid binding, and disruption of protein folding, allowing AMPs to exhibit bactericidal effects against ESBL-producing Enterobacterales, CRE, and *B. fragilis* [64]. These peptides, such as human cathelicidin LL-37 and defensins, and synthetic derivatives, such as the peptide SAAP-148, have shown efficacy against biofilm-associated infections caused by MDR *Pseudomonas aeruginosa* and *Enterococcus faecium* in preclinical wound and peritonitis models [64,65]. Clinical transposition of AMPs has been hampered by challenges such as susceptibility to proteolytic degradation in biological fluids, potential toxicity at higher concentrations, and high production costs. Strategies to overcome these limitations include peptidomimetic engineering, d-amino acid substitutions, cyclization, and nanoparticle-based delivery systems that protect AMPs from enzymatic degradation while enhancing their local bioavailability at intra-abdominal sites of infection [63,66].

#### 2.3.3. Bacteriophage Therapy

Bacteriophages are viruses that exclusively infect and lyse bacterial cells, offering a highly specific antimicrobial strategy. Their relevance has increased considerably in scientific and clinical communities, particularly in the context of antimicrobial resistance. Bacteriophage therapy offers several theoretical advantages for IAI: exceptional specificity at a species and strain level, which spares the commensal microbiota; self-amplifying pharmacokinetics at the site of infection; efficacy against organisms incorporated into the biofilm; and activity independent of conventional mechanisms of antibiotic resistance [67,68]. There is substantial preclinical evidence in support of bacteriophage therapy in IAI. Cocktails targeting *Pseudomonas*, *Acinetobacter baumannii*, *K. pneumoniae*, and MDR *E. coli* demonstrated significant reductions in bacterial load and improved survival in murine models of peritonitis and intra-abdominal sepsis [67,68,69].

Key challenges for bacteriophage therapy in IAI include the narrow host range of individual phages (which requires personalized or combined approaches), the potential for rapid bacterial resistance through receptor mutation, the complexity of biologic therapy regulations, and the lack of standardized pharmacokinetic and pharmacodynamic frameworks. Genetically modified phages with a wide range of hosts and phage-antibiotic synergy (PAS) combinations are promising strategies that are currently under active investigation [68,70].

#### 2.3.4. CRISPR/Cas Systems

The CRISPR (Clustered Regularly Interspaced Short Palindromic Repeats)/Cas system, initially characterized as an adaptive immune mechanism in prokaryotes, has been reused as a precision tool to combat antimicrobial resistance. CRISPR-based antimicrobial strategies exploit programmable guide RNA to direct Cas nuclease (usually Cas9 or Cas13) to cleave specific DNA or RNA sequences in bacterial cells, allowing for specific killing of targeted pathogen sequences or selective elimination of resistance genes [71].

Two main applications are relevant to AMR in IAI. First, CRISPR/Cas systems can be administered through bacteriophages or conjugative plasmids to selectively destroy bacteria carrying specific determinants of resistance (e.g., *blaKPC*, *blaNDM*, or *vanA* genes), thereby sensitizing populations resistant to conventional antibiotics [17,18]. Second, CRISPR-based diagnostics (e.g., SHERLOCK, DETECTR) allow for rapid and ultrasensitive detection of resistance genes directly from clinical samples, facilitating the early identification of MDR pathogens in peritoneal fluid and aspirates from abscesses [72].

Although CRISPR-based antimicrobials are largely in the preclinical stage, proof-of-concept studies have demonstrated successful elimination of carbapenem-resistant *E. coli* in vivo in murine models of colonization and intestinal infection using phage-delivered CRISPR/Cas9 constructs [71]. Significant barriers to clinical application include the efficient and targeted delivery of CRISPR components to sites of polymicrobial infections, off-target effects, horizontal gene transfer of CRISPR elements, immune responses to Cas proteins, and the complex regulatory pathway for gene editing therapies [72,73].

#### 2.3.5. Probiotics and Microbiome-Based Strategies

The gut microbiome plays a central role in both the pathogenesis and treatment of IAI. Disruption of commensal microbial communities (dysbiosis), often precipitated by broad-spectrum antibiotic therapy, facilitates the overgrowth and translocation of MDR pathogens, thereby increasing the risk of recurrent or persistent IAI [74]. Probiotic and microbiome-based interventions aim to restore microbial homeostasis, strengthen resistance to colonization against MDR organisms, and modulate local and systemic immune responses.

Specific strains of *Lactobacillus*, *Bifidobacterium* and *Saccharomyces boulardii* have demonstrated the ability to inhibit the growth and adhesion of MDR species *E. coli*, *K. pneumoniae* and *Enterococcus* spp. by competitive exclusion, production of bacteriocins and organic acids and improvement of intestinal barrier function [74]. Perioperative supplementation with probiotics in patients undergoing abdominal surgery reduces the incidence of infections at the site of surgery and postoperative intra-abdominal complications, including peritonitis associated with anastomotic fistulas [75,76].

Emerging microbiome-based therapies, including FMT, and commensal bacteria consortia have proven effective in decolonizing the gut of MDR organisms, especially CRE and VRE (Vancomycin-Resistant *Enterococci*), potentially reducing the reservoir of resistant pathogens that cause secondary IAI [74,77]. In 2023, the FDA approved the first orally administered fecal microbiota product, which contains live spores and is intended for the prevention of recurrent CDI [78]. However, concerns about the safety of administration of live organisms to immunocompromised or critically ill surgical patients, as well as the need for strain-specific evidence of efficacy, require prudent clinical implementation.

## 3. Discussion

The relentless advance of antimicrobial resistance among pathogens causing s demands a comprehensive, multi-pronged strategy that integrates both optimization of the conventional antibiotic pipeline and the development of non-traditional antimicrobial modalities.

The recent approvals of novel BL/BLI combinations have markedly expanded the therapeutic armamentarium against KPC-producing CRE, CRAB, and MDR *P. aeruginosa* in cIAIs [26,34,79,80]. The emergence of next-generation combinations targeting metallo-beta-lactamases, particularly cefepime/taniborbactam and cefepime/zidebactam, represents a critical step toward closing one of the most significant remaining gaps in coverage against MBL-producing organisms, though their IAI-specific indications await further Phase 3 data [81,82]. The novel approach of pairing cefiderocol with the pan-spectrum boronate inhibitor xeruborbactam signals a further evolution in BL/BLI design, potentially addressing even double-carbapenemase-producing Enterobacterales [83].

Nonetheless, the conventional pipeline alone is insufficient to outpace the accelerated evolution of resistance. The WHO’s 2025 analysis underscored that most pipeline agents represent iterative improvements upon existing scaffolds for which resistance mechanisms are already well established, and that truly innovative agents with novel mechanisms remain scarce. Furthermore, the challenges of antibiotic market economics continue to threaten the sustainability of even successful novel agents, as evidenced by the bankruptcy or financial distress of several small pharmaceutical companies that brought innovative antibiotics to market [39,84,85].

In this context, the five non-traditional strategies reviewed in this paper, nanoparticles, antimicrobial peptides, bacteriophages, CRISPR/Cas systems, and probiotics, offer distinct and complementary mechanisms that circumvent established resistance pathways through fundamentally different modes of action. Their convergence with conventional antibiotic therapy into combination or sequential treatment strategies holds promise. For instance, phage-antibiotic synergy (PAS), in which lytic bacteriophages are co-administered with sub-inhibitory concentrations of beta-lactams to enhance bacterial killing and suppress resistance emergence, could be combined with newly approved BL/BLI agents to create synergistic therapeutic frameworks [70,86]. Similarly, CRISPR-based re-sensitization of CRE strains carrying *bla*NDM or *bla*KPC genes, followed by targeted therapy with cefepime/taniborbactam or aztreonam/avibactam, represents a conceptually compelling strategy for overcoming even pan-resistant infections [87,88]. Nanoparticle-based drug delivery systems offer the potential to enhance the peritoneal bioavailability and reduce the systemic toxicity of both conventional and novel antibiotics, while microbiome-based strategies, including targeted probiotics and FMT, can complement antimicrobial therapy by restoring colonization resistance and reducing the selective pressure that drives resistance amplification in the gut reservoir [89,90]. The integration of these approaches with rapid molecular diagnosis such as metagenomic next-generation sequencing and multiplex PCR panels that enable same-day pathogen and resistome identification will be essential for implementing precision antimicrobial strategies in cIAI management.

Looking ahead, several critical research priorities must be addressed to translate these promising strategies into clinical practice for cIAI patients. Large-scale, prospective, randomized controlled trials evaluating the clinical impact of non-traditional antimicrobials (particularly phage therapy and AMPs) in cIAI-specific populations are urgently needed. Pharmacokinetic and pharmacodynamic studies of novel agents within the peritoneal compartment, a pharmacologically distinct environment characterized by variable protein binding, pH fluctuations, and fibrin deposition, are essential for optimizing dosing strategies. Regulatory frameworks for living biological therapeutics (phages, CRISPR constructs) and adaptive trial designs that accommodate personalized approaches must be developed. Finally, equitable global access to both next-generation BL/BLI combinations and non-traditional antimicrobials will require sustained public and private investment, international coordination, and innovative economic models that decouple antibiotic revenue from volume of use.

A summary of selected antimicrobial agents and non-antibiotic strategies according to drug class and development or approval status is presented in Table 1 and Table 2. Agents are categorized as preclinical/experimental, Phase I, Phase II, Phase III/regulatory, approved for cIAI, or approved for non-IAI indications with potential translational relevance. CRAB-directed agents are included because carbapenem-resistant *Acinetobacter baumannii* may contribute to healthcare-associated or postoperative IAI, although most CRAB pipeline agents are not IAI-specific or IAI-approved.

## 4. Materials and Methods

A comprehensive literature search was conducted to identify relevant preclinical, translational, and clinical evidence regarding novel antimicrobial agents, as well as alternatives including nanoparticles, antimicrobial peptides, bacteriophages, CRISPR/Cas systems, and probiotics, in the context of antimicrobial resistance among pathogens implicated in intra-abdominal infections. Electronic databases searched included PubMed/MEDLINE, Scopus, Web of Science, and the Cochrane Library. The search was limited to articles in English, covering publications from January 2014 through December 2025, to capture the most recent decade of evidence in this rapidly evolving field. We also screened Google Scholar for initial scoping and comprehensive citation tracking.

Structured search queries were constructed using Medical Subject Headings (MeSH) terms and free-text keywords combined with Boolean operators. The primary search string incorporated the following domain-specific term sets: (a) infection: “intra-abdominal infection” OR “peritonitis” OR “intra-abdominal abscess” OR “intra-abdominal sepsis” OR “surgical site infection” OR “abdominal sepsis”; (b) resistance: “antimicrobial resistance” OR “multidrug-resistant” OR “ESBL” OR “carbapenem-resistant” OR “VRE” OR “CRE” OR “MDR”; and (c) intervention: “nanoparticles” OR “silver nanoparticles” OR “antimicrobial peptides” OR “bacteriophage therapy” OR “phage therapy” OR “CRISPR” OR “CRISPR-Cas” OR “probiotics” OR “microbiome therapy”. These three term sets were combined using the AND operator to ensure retrieved articles addressed the intersection of all three domains.

Studies were eligible for information screening if they met the following criteria: (a) original research articles (in vitro, in vivo, or clinical studies), systematic reviews, meta-analyses, or narrative reviews addressing one or more of the target antimicrobial strategies; (b) focus on bacterial pathogens of established relevance to IAIs, including Enterobacterales (*E. coli*, *K. pneumoniae, Enterobacter* spp.), *Enterococcus* spp., *Bacteroides fragilis*, *Pseudomonas aeruginosa*, and *Acinetobacter baumannii*; and (c) explicit evaluation of antimicrobial activity against drug-resistant strains or discussion of resistance mechanisms. Case reports with fewer than three patients (unless representing first-in-human reports), conference abstracts without full-text availability, studies focused exclusively on non-abdominal infection sites without generalizable mechanistic data, and publications in non-peer-reviewed sources were excluded from this review analysis.

Titles and abstracts of retrieved records were screened for relevance. Full-text articles meeting the eligibility criteria were then assessed in detail. Reference lists of included systematic reviews and key narrative reviews were hand-searched to identify additional pertinent studies not captured by the electronic search.

**Figure 1:**
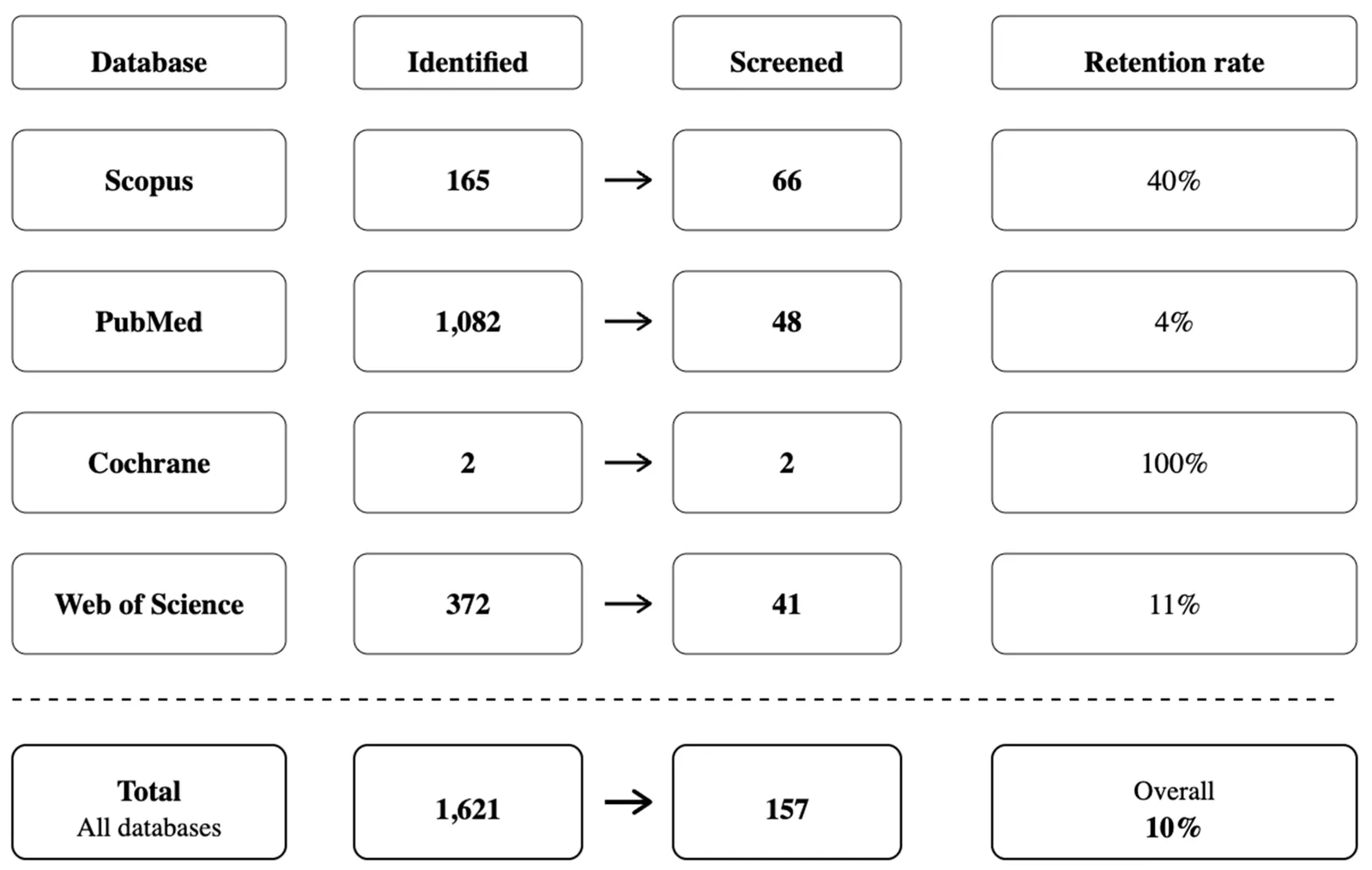
Flow diagram of database search results and retention rates.

From 1621 identified records across all databases, only 157 (10%) proceeded to screening, and 46 studies were included in the analysis, while additional data was extracted from references and from other sources (WHO, FDA, EMA). Given the heterogeneity of study designs, model systems, and intervention types across therapeutic domains, a formal systematic review and quantitative meta-analysis was not feasible. Instead, a narrative synthesis approach was adopted, in which findings were organized thematically by antimicrobial strategy and contextualized within the clinical framework of AMR in cIAIs. A flow diagram of the study identification and screening process can be found in Figure 1.

Identified: records returned by database search; Screened: records assessed for eligibility; Retention rate: percentage of identified records screened.

## 5. Conclusions

In conclusion, the convergence of novel antibiotic agents, particularly the expanding portfolio of BL/BLI combinations with activity against MBL-producing organisms and the five non-traditional antimicrobial strategies reviewed herein provides the foundation for a new paradigm in the management of drug-resistant intra-abdominal infections. The transition from a monotherapy-centric approach to an integrated, multi-modal treatment framework, guided by rapid diagnostics and informed by antimicrobial stewardship, will be essential to preserve therapeutic efficacy against the most formidable AMR threats of the coming decades.

## 6. Limitations

This study was designed as a focused, expert narrative review rather than a formal systematic review with PRISMA-compliant reporting. As such, the selection of studies was guided by relevance, methodological quality, and recency rather than exhaustive enumeration. The rapidly evolving nature of several of these fields, particularly CRISPR-based antimicrobials and clinical phage therapy, means that new evidence may emerge between the time of writing and publication.

Secondly, the scope of this review is necessarily limited to intra-abdominal infections and may not fully capture the broader context of antimicrobial resistance or the relative merits of these approaches for other infection types where evidence may be more developed. Where available, data from cIAI-specific studies were prioritized. However, given the relative scarcity of cIAI-focused trials for several strategies, high-quality evidence from related infection models (bloodstream infections, wound infections, respiratory tract infections) was included when mechanistic principles were generalizable to the intra-abdominal compartment. The review focuses primarily on published clinical research and established animal models, potentially underrepresenting important negative studies or failed clinical programs that would provide valuable context regarding the limitations of these approaches.

Additionally, economic analyses are limited and often derived from preliminary cost estimates rather than real-world manufacturing data; actual costs at clinical scale remain uncertain and may differ substantially from projections. This review does not provide detailed pharmacoeconomic analysis comparing cost-effectiveness of novel approaches versus conventional antibiotics in various clinical scenarios.

Third, potential author bias should be acknowledged. Although this review aims to balance promise and limitations, the authors’ professional perspectives may shape interpretation, and clinicians in infectious diseases, microbiology, or antimicrobial stewardship may weigh these limitations differently. The review also does not comprehensively address very early-stage technologies, such as immunophages, engineered phage-antibiotic combinations, or synthetic biology approaches, some of which may ultimately prove highly relevant. Its cautious emphasis on limitations may underestimate technologies still in development, while discussion of promising preclinical results may overstate the likelihood of clinical translation. Finally, regulatory and reimbursement differences across regions, which may strongly affect access, are not addressed in detail.

## Figures and Tables

**Table 1:** Antimicrobial agents and strategies approved for or potentially translatable to IAIs.

Drug Class	Agent	Class/Mechanism	Approved Indication(s)	FDA Approval
β-Lactam/β-lactamase inhibitor (BLI) combinations	Ceftolozane/tazobactam	Cephalosporin + BLI	cIAI (with metronidazole); also cUTI, HABP/VABP	2014
	Ceftazidime/avibactam	Cephalosporin + BLI	cIAI (with metronidazole); also cUTI, HABP/VABP	2015
	Imipenem/cilastatin/relebactam	Carbapenem + BLI	cIAI; also cUTI, HABP/VABP	2019
	Aztreonam/avibactam	Monobactam + BLI	cIAI (with metronidazole) *	2025
	Meropenem/vaborbactam	Carbapenem + BLI	cUTI	2017
	Cefepime/enmetazobactam	Cephalosporin + BLI	cUTI	2024
	Sulbactam/durlobactam	Sulbactam + BLI	HABP/VABP due to Acinetobacter (CRAB)	2023
	Cefepime/zidebactam	Cephalosporin + β-lactam enhancer	cUTI	2026
Other β-lactams/ related agents	Cefiderocol	Siderophore cephalosporin	cUTI; HABP/VABP	2019
	Ceftobiprole medocaril	Anti-MRSA cephalosporin	SAB; ABSSSI; CABP	2024
	Sulopenem etzadroxil/ probenecid	Oral penem	uUTI	2024
Fluorocyclines/ tetracyclines	Eravacycline	Fluorocycline	cIAI	2018
Aminoglycosides	Plazomicin	Aminoglycoside	cUTI	2018
Novel fluoroquinolones	Delafloxacin	Fluoroquinolone	ABSSSI; CABP	2017
Non-fluoroquinolone topoisomerase inhibitors	Gepotidacin	Triazaacenaphthylene topoisomerase inhibitor	uUTI; uncomplicated urogenital gonorrhoea	2025
Non-classical adjuncts	Fecal microbiota spores, live-brpk	Oral microbiome therapeutic	Prevention of recurrent CDI	2023

* Restricted to patients who have limited or no alternative treatment options. ABSSSI, acute bacterial skin & skin-structure infection; BLI, β-lactamase inhibitor; CABP, community-acquired bacterial pneumonia; CDI, *Clostridioides difficile* infection; cIAI, complicated intra-abdominal infection; CRAB, carbapenem-resistant *Acinetobacter baumannii*; cUTI, complicated urinary-tract infection; HABP, hospital-acquired bacterial pneumonia; IAI, intra-abdominal infection; MRSA, methicillin-resistant *Staphylococcus aureus*; SAB, *Staphylococcus aureus* bacteraemia; uUTI, uncomplicated urinary-tract infection; VABP, ventilator-associated bacterial pneumonia.

**Table 2:** Pipeline agents and non-traditional adjunctive strategies under investigation in or potentially translatable to IAIs.

Drug Class	Agent	Highest Development Stage
β-Lactam/BLI combinations	Xeruborbactam (QPX-7728) [91]	Phase I
	Meropenem—ANT3310 [92]	Phase I
	Cefiderocol/xeruborbactam [34]	Phase I
	Cefepime—taniborbactam (cUTI) [93]	Phase III/Regulatory
	Cefepime/nacubactam (cUTI) [94]	Phase III/Regulatory
	Aztreonam/nacubactam (cUTI) [94]	Phase III/Regulatory
	Imipenem/cilastatin/funobactam (cUTI) [95]	Phase III/Regulatory
Other β-lactams/related agents	Cefiderocol/taniborbactam [40]	Preclinical (in vitro only)
	Sulopenem—iv formulation * [48]	Phase III/Regulatory
Fluorocyclines/tetracyclines	Zifanocycline (KBP-7072) [27]	Phase I
Aminoglycosides	Apramycin (EBL-1003) [96]	Phase I
Polymyxin analogues/lipopeptides [27]	Upleganan (SPR206/EVER206)	Phase I
	MRX-8	Phase I
	QPX-9003	Phase I
CRAB-focused novel classes	R-327 [27]	Phase I
	Zosurabalpin (RG6006) [27]	Phase I
	BV-100 [97]	Phase II
	OMN-6 [27]	Phase II
	Cefepime/Zidebactam (WCK-5222) † [27]	Phase III/Regulatory
	Funobactam (XNW-4107) [27]	Phase III/Regulatory
Novel fluoroquinolones	Avarofloxacin (JNJ-Q2)—ABSSSI [98,99]	Phase II
	Finafloxacin—cUTI [100]	Phase II
Non-classical adjuncts (all preclinical/experimental)	Metal & metal-oxide nanoparticles (Ag, ZnO, CuO)	Preclinical/Experimental
	Polymeric nanoparticles (chitosan, PLGA)	Preclinical/Experimental
	PEG-PPS and PVA-vancomycin surface coatings	Preclinical/Experimental
	Silver-nanoparticle screws; PLGA bone grafts	Preclinical/Experimental
	Antimicrobial peptides (cathelicidin LL-37, defensins, SAAP-148)	Preclinical/Experimental
	Bacteriophage therapy; CRISPR/Cas antimicrobials	Preclinical/Experimental
	Probiotics/postbiotics/synbiotics	Preclinical/Experimental
	Toxin–antitoxin systems	Preclinical/Experimental

* Oral sulopenem (etzadroxil/probenecid, Orlynvah) is FDA-approved for uUTI (Table 1); the intravenous formulation for cIAI remains in development. The Phase 3 cIAI trial failed to meet FDA-specified primary endpoint (non-inferiority to ertapenem in the micro-modified intent-to-treat population). † Zidebactam is the β-lactam “enhancer” in cefepime/zidebactam (WCK 5222). The combination is FDA-approved for cUTI (2026; Table 1); it remains investigational for other indications including CRAB.

## Data Availability

No new data were created or analyzed in this study. Data sharing is not applicable.

## Abbreviations

The following abbreviations are used in this manuscript:

ABSSSI	Acute bacterial skin and skin-structure infection
AgNPs	Silver nanoparticles
ALIF	Anterior lumbar interbody fusion
AMPs	Antimicrobial peptides
AMR	Antimicrobial resistance
*bla*	β-lactamase gene prefix
*blaCTX-M*	CTX-M-type extended-spectrum β-lactamase gene
*blaIMP*	Imipenemase metallo-β-lactamase gene
*blaKPC*	Klebsiella pneumoniae carbapenemase gene
*blaNDM*	New Delhi metallo-β-lactamase gene
*blaOXA-23-like*	OXA-23-like oxacillinase carbapenemase gene
*blaOXA-48-like*	OXA-48-like oxacillinase carbapenemase gene
*blaSHV*	Sulfhydryl-variable β-lactamase gene
*blaTEM*	TEM-type β-lactamase gene
*blaVIM*	Verona integron-encoded metallo-β-lactamase gene
BL/BLI	Beta-lactam/beta-lactamase inhibitor
BLI	β-lactamase inhibitor
CABP	Community-acquired bacterial pneumonia
CDI	Clostridioides difficile infection
*cepA*	Chromosomal cephalosporinase gene in *Bacteroides* spp.
*cfiA*	Carbapenemase gene in the *Bacteroides fragilis* group
*cfxA*	β-lactamase gene in anaerobic bacteria
cIAI	Complicated intra-abdominal infection
CRAB	Carbapenem-resistant *Acinetobacter baumannii*
CRE	Carbapenem-resistant Enterobacterales
CRISPR	Clustered Regularly Interspaced Short Palindromic Repeats
CRPA	Carbapenem-resistant *Pseudomonas aeruginosa*
CuO	Copper oxide
cUTI	Complicated urinary tract infection
DDI	Drug–drug interaction
ERG11	Lanosterol 14-α-demethylase gene associated with azole resistance in *Candida* spp.
*ermF*	Erythromycin ribosomal methylase gene associated with macrolide–lincosamide resistance
ESBL	Extended-spectrum β-lactamase
FKS1/FKS2	Genes encoding echinocandin target subunits of β-1,3-D-glucan synthase
FMT	Fecal microbiota transplantation
GU infection	Genitourinary infection
HABP	Hospital-acquired bacterial pneumonia
IAI	Intra-abdominal infection
ICU	Intensive care unit
IMP	Imipenemase metallo-β-lactamase
KPC	*Klebsiella pneumoniae* carbapenemase
LIF	Lateral interbody fusion
LL-37	Human cathelicidin antimicrobial peptide LL-37
LPS	Lipopolysaccharide
MBL	Metallo-β-lactamase
MDR	Multidrug-resistant
*mecA/mecC*	Genes encoding altered penicillin-binding protein PBP2a/PBP2c in methicillin-resistant staphylococci
MRSA	Methicillin-resistant *Staphylococcus aureus*
MTZ	Metronidazole
NDM	New Delhi metallo-β-lactamase
*nim*	Nitroimidazole resistance gene associated with metronidazole resistance
NPs	Nanoparticles
OLIF	Oblique lateral interbody fusion
*oprD*	Outer membrane porin D gene in *Pseudomonas aeruginosa*
PAS	Phage-antibiotic synergy
PBP2	Penicillin-binding protein 2
PDR	Pan-drug resistant
PEG-PPS	Poly(ethylene glycol)-poly(propylene sulfide)
PLGA	Poly(lactic-co-glycolic acid)
PVA	Poly(vinyl alcohol)
ROS	Reactive oxygen species
SAB	*Staphylococcus aureus* bacteremia
spp.	Species pluralis
SSI	Surgical site infection
*tetQ*	Tetracycline resistance gene
uUTI	Uncomplicated urinary tract infection
VABP	Ventilator-associated bacterial pneumonia
VIM	Verona integron-encoded metallo-β-lactamase
VRE	Vancomycin-resistant enterococci
WHO	World Health Organization
XLIF	Extreme lateral interbody fusion
XDR	Extended-drug resistant
ZnO	Zinc oxide
